# Origins of life: the possible and the actual

**DOI:** 10.1098/rstb.2024.0281

**Published:** 2025-10-02

**Authors:** Ricard Solé, Christopher Kempes, Susan Stepney

**Affiliations:** ^1^ Complex Systems Lab, Universitat Pompeu Fabra, Dr Aiguader 88, 08003 Barcelona, Spain; ^2^ Institució Catalana de la Recerca i Estudis Avançats (ICREA), Passeig Lluıs Companys 23, 08010 Barcelona, Spain; ^3^ Institut de Biologia Evolutiva, CSIC-UPF, Pg Maritim de la Barceloneta 37, 08003 Barcelona, Spain; ^4^ Santa Fe Institute, 1399 Hyde Park Road, Santa Fe, NM 87501, USA; ^5^ Department of Computer Science, University of York, Deramore Lane, York YO10 5GH, UK

**Keywords:** origins of life, geochemistry, artificial life, synthetic biology, evolution, complexity

## Abstract

The questions of how life forms, whether life is an inevitable outcome and how diverse its presentation could be remain some of the most profound in science. Investigations into the origin of life confront key issues such as uncovering key constraints and universal features of life, the plausibility of alternative biochemistries and the transition from purely chemical systems to information-bearing, evolvable entities. Many of these issues can be associated with early cell formation and evolution. Thus, protocellular systems have emerged as a key focus of study. Here, the community can ask questions about physical constraints and the co-evolution of energy, matter and information. The pursuit of these answers spans a wide range of disciplines, including geochemistry, statistical physics, systems and evolutionary biology, artificial life, synthetic biology and information theory, and reflects the inherently interdisciplinary nature of origin-of-life research. This article surveys key theoretical frameworks and experimental approaches that have shaped our current understanding, while outlining the major unresolved challenges that continue to drive the field forward. It also summarizes and contextualizes the articles in this special issue that address these questions.

This article is part of the theme issue ‘Origins of life: the possible and the actual’.

## 1. Introduction

Is life a rare, perhaps improbable event in the universe? Is it the outcome of a sequence of fortunate accidents tightly constrained by planetary conditions and the chemistry of its environment? Or is it an almost inevitable outcome of complex geochemistry and deep time? As we seek to understand how life emerged on Earth, we also face broader questions about the likelihood of life elsewhere. Can the study of our own biosphere, together with growing knowledge of our solar system and the discovery of thousands of exoplanets, offer clues about the conditions necessary for life to arise and evolve? Could there be multiple pathways through which life could have taken hold, some potentially diverging dramatically from the biochemical framework with which we are familiar [1–4]? Is life necessarily tied to information processing, replication and the principle of natural selection, or could other organizing principles give rise to living systems [5,6]? Furthermore, can advances in synthetic biology and artificial life—whether created *in vitro* in the lab or modelled *in silico* through computational simulations—reveal the generative mechanisms that underpin life itself [7,8]? These questions lie at the heart of one of the most profound frontiers of science: understanding the origins, nature and potential ubiquity of life in the cosmos.

The questions outlined above have been subjects of intense inquiry since the origin of life (OOL) became a recognized scientific problem. This transition, from philosophical speculation to empirical investigation, gained momentum during the mid−twentieth century and was often considered the golden age of OOL research. The early work of Oparin on prebiotic systems [9,10] and the groundbreaking experiments by Miller in 1953 on amino acid synthesis ([11]; see also Bada & Lazcano [12]] and Oró on nucleobase synthesis [13,14], laid the foundation for modern studies, and other scholars expanded on the initial concepts [15]. It was soon understood that the chemical scenario also required an understanding of the planetary context. Comets were soon proposed as candidates for the transport of extraterrestrial organic matter to Earth [16–19]. Their work marked a turning point: life’s beginnings could now be explored through chemistry, biology and planetary science, opening the door to rigorous hypotheses about how living systems could emerge from nonliving matter.

**Figure 1.**
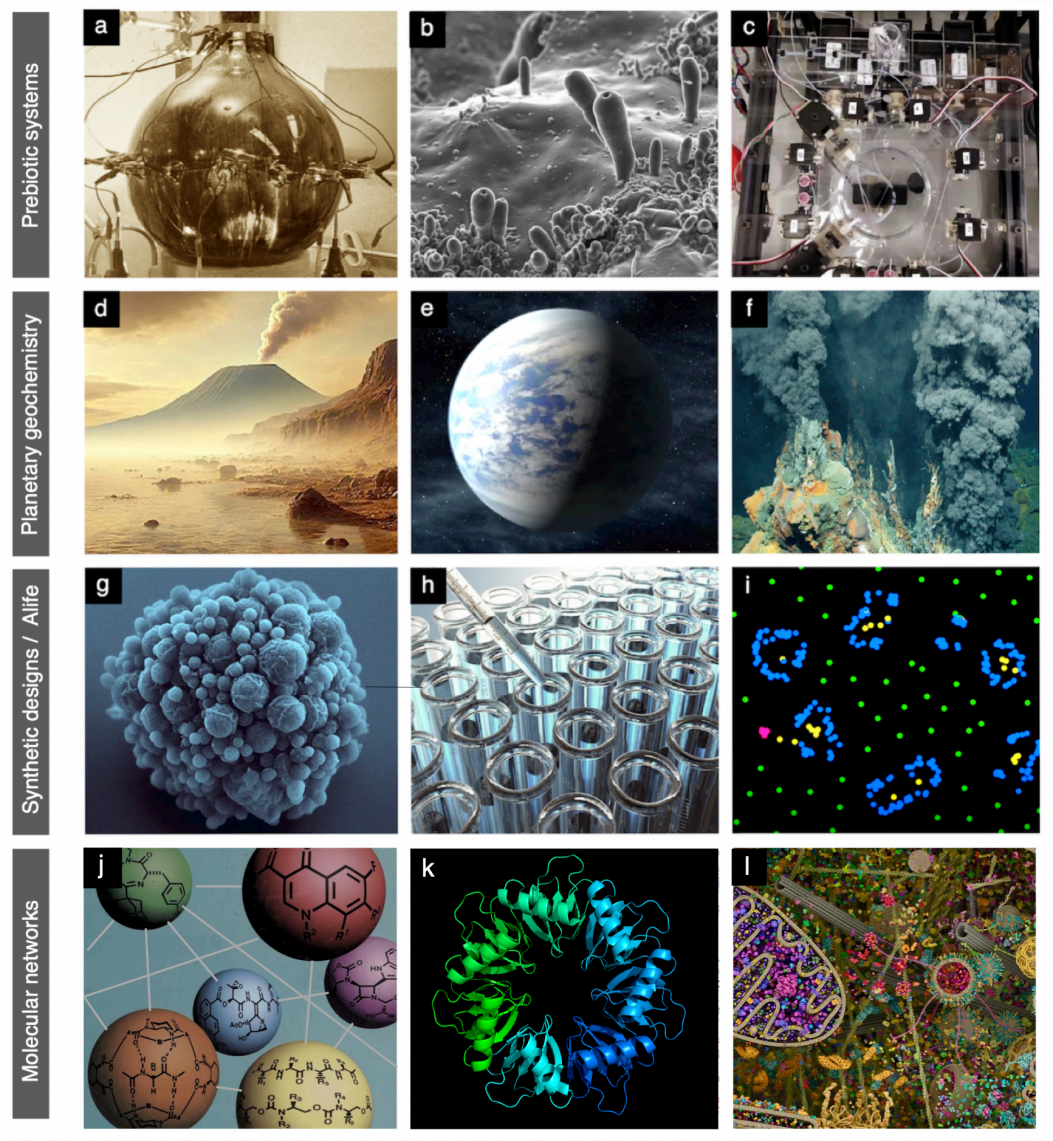
The many paths to the OOL. Several approaches to the problem of how life originated, evolved and became complex are outlined here. These include, among others: (a) prebiotic experiments using simple mixtures of molecules, such as this recreation of Titan's atmosphere; (b) extended Miller-like experiments [11] with concomitant formation of embodied structures; (c) evolutionary dynamics of chemical droplets using a combination of *in vitro* selection and AI; (d) the analysis of primitive Earth environments; (e) the exploration of exoplanets; (f) extreme life forms, such as those found in ocean vents; (g) engineering minimal genomes, such *Mycoplasma* JVC1-syn1.0; (h) cell-free systems obtained from cells or reconstituted from purified components and (i) spatially extended artificial life models (here Lenia [22,23]); (j) combinatorial chemistry studies; (k) evolutionary aspects of metabolic networks and the diverse components associated to them, inferred from comparative genomics studies; and (l) full-model simulation of cells using (still limited) computational approximations to molecular dynamics that try to replicate (on short time scales) some of the supramolecular processes taking place in the crowded cellular environment (illustration by D. Goodsell).

Building on the recognition that life’s origin poses a multifaceted scientific challenge, it becomes clear that any meaningful progress requires navigating a diverse landscape of theoretical approaches and empirical domains [20,21]. As summarized in figure 1, this landscape spans multiple scales, from planetary and geochemical constraints to molecular evolution, combinatorial chemistry and the emergence of metabolic organization. Research efforts increasingly draw on a broad toolkit, incorporating insights from extreme environments, exoplanet studies, prebiotic synthesis experiments and synthetic biology.

These diverse lines of inquiry have fuelled a growing synergy between empirical findings and theoretical or computational modelling. Such cross-disciplinary efforts are essential to identify the general conditions under which life-like behaviour might arise, both on Earth and in other planetary settings [24–26]. In this regard, the planetary context provides a natural framework: from interpreting atmospheric biosignatures on exoplanets [27,28], to modelling chemical reaction networks in early Earth and interstellar environments [29–32].

Importantly, as in other domains of evolutionary biology, effective theories of the emergence of life must reconcile historical contingency with the restrictions imposed by physical laws [33]. The convergence of classical hypotheses with contemporary advances in systems chemistry, planetary science and network theory has coalesced in the field of astrobiology, a domain uniquely positioned to explore life as a planetary and universal phenomenon [34]. Since terrestrial life emerged in our remote past, a direct experimental testing of such a deep-time event needs to be replaced by alternatives. As mentioned above, prebiotic chemistry offered the first approach to this problem, and in recent decades novel experimental implementations, including evolutionary dynamics and Darwinian selection [35,36], have been developed. Within the field of artificial life, multiple paths have been explored to investigate this concept, including artificial chemistries [37].

In 2021, the Santa Fe Institute hosted a workshop aimed at addressing fundamental questions about the possible pathways and actual processes underlying the OOL. The meeting brought together researchers from a broad spectrum of disciplines, including thermodynamics, information theory and synthetic biology, to explore the multifaceted nature of this problem. This Theme Issue presents a synthesis of key ideas that emerged from the discussions, highlighting the main challenges in understanding the origins of life. These range from theoretical and conceptual frameworks to planetary-scale environmental considerations, as well as experimental efforts to recreate life-like systems in the laboratory.

## 2. What is life? Is it expected?

If we want to understand life, should we not first have an operational definition of it [3,6,8]? This seems like a reasonable question, since assessing the likelihood of life’s existence requires first knowing how to recognize it. However, despite our extensive knowledge of living systems, we are still far from reaching a consensus. With regard to this problem, the question arises as to how to distinguish between animate and inanimate systems when considering their change and evolution. Recently, methods for measuring the complexity of any molecule have been proposed^1^ [42,43], with interesting discussions about minerals [44,45], which have been a complicated case for OOL research for some time [46,47]. For living matter, there is general agreement that it would follow natural selection dynamics. We understand well how this theory works and how to define rigorous mathematical models [48]. Although the scope of evolution has at times been extended well beyond biology, it has raised contentious debates about its applicability. Cairns-Smith, for example, proposed that life on Earth originated through a process of natural selection that acts on inorganic crystals [49], while Pross has explored the possibility of applying Darwinian principles to inanimate physicochemical systems [50]. Although provocative, such views remain problematic because they rely on substrates fundamentally distinct from living matter and lack the organizational features, such as heredity, variation and regulated metabolism, that underpin Darwinian evolution in biological systems. As such, these approaches provide, at best, useful metaphors rather than plausible mechanisms for the actual origins of life.

The problem of defining life has deep historical roots, tracing back to ancient philosophy when thinkers like Aristotle sought to distinguish living from nonliving based on characteristics such as self-movement [51], growth and reproduction. With the rise of modern science, especially after the advent of cell theory and Darwinian evolution, biological definitions emphasized structural and functional criteria, such as metabolism and natural selection. In the twentieth century, new perspectives emerged from cybernetics, systems theory and information science, introducing concepts such as homoeostasis [52], autopoiesis [53] and informational self-replication. Despite these advances, each disciplinary lens captured only part of the phenomenon, leading to a proliferation of definitions without consensus. The challenge has intensified in recent decades with developments in synthetic biology, artificial life and astrobiology, which increasingly blur the boundaries of what qualifies as ‘alive’ [54] and expose the limitations of traditional biological definitions [55]. In [56], the authors have examined the difficulties inherent in evaluating speculative hypotheses about the OOL (what they call the Science of the Gaps), emphasizing how complexity and historical contingency create persistent gaps in both knowledge and conceptual frameworks. Along with the limitations imposed by current empirical approaches, metaphors and biased narrative scenarios have played an important role. By formalizing causal assumptions, they propose a more systematic approach to reasoning through these gaps, enabling a more rigorous evaluation of fragmentary and speculative OOL models.

Scholars hold differing views on the inevitability of life. Some, like de Duve, argue that life is a cosmic imperative, suggesting that, given the right conditions, life will emerge as a natural consequence of chemistry [57]. Others, such as Monod, emphasize the role of chance and contingency, viewing life as a highly improbable event in the universe [58]. Still others, including proponents of systems chemistry and self-organization, propose that life arises from the inherent combinatorial dynamics of complex systems, making it statistically likely under certain constraints [59,60]. This ongoing debate reflects deeper philosophical divisions about whether life is an accident or an expected outcome of physical laws. An important component in this context is the role that the combinatorial potential of living objects plays, particularly at the molecular level. It is well known that the dynamics of large collections of objects capable of assembling or combining can easily drive a hyperbolic growth process [43,61–64]. How does this accelerated growth dynamics impact evolution and its predictability?

The combinatorial nature of evolution [65] has been argued to pose a fundamental challenge to the formulation of a predictive theory of evolutionary change (see also this problem within the context of cellular automata and computation [66–70]). This difficulty is made explicit through the concept of the *adjacent possible*. Briefly, the adjacent possible refers to the set of novel biological forms or functions that can emerge through small, incremental modifications of a system’s current state. Evolutionary innovations emerge from this constrained space of possibilities, and as new structures or functions are realized, the adjacent possible itself expands, enabling further exploration. According to Kauffman, this process is not algorithmically predictable: we cannot prestate the full repertoire of potential evolutionary trajectories or innovations, since the space of possibilities evolves in tandem with the system. Using the concept of the Adjacent Possible, Kauffman & Roli [71] propose that life is an expected consequence of the (first-order) transition from random reaction chemistry to autocatalytic chemical sets (see [72]) along with RNA sets displaying catalytic properties. These two sets would eventually cooperate to form some class of minimal (cellular) agent capable of template replication and coding.

## 3. Universal constraints

A key focus for OOL research is identifying the aspects of life that can be counted on to be universal [3,6]. Much discussion has been given to the material aspects of life, where debates about the centrality of carbon and a water solvent have occurred for decades [1,73,74]. The broader question here is how can we draw general inferences about origins of life, given the immense span of time since it arose and the potentially contingent nature of the processes involved? This challenge is closely linked to the historical character of molecular evolution: did life on Earth—and possibly elsewhere—emerge through multiple distinct pathways? [3,4] Alternatively, as some studies propose, might there be universal properties of complex systems that constrain and shape the range of possible outcomes, or clear signatures of those outcomes [6,43]? Given that we possess only a single large-scale natural instance of life’s emergence—our biosphere—the lack of comparative data may seem to preclude meaningful inference.

One way to overcome this problem is to take lessons from physics, particularly cosmology. Cosmology exemplifies the power of physics-based science to probe the deep past through indirect but quantitatively robust evidence. Despite dealing with phenomena occurring billions of years ago, the standard cosmological model achieves predictive accuracy by anchoring itself in general relativity, quantum field theory and thermodynamics. Observables such as the cosmic microwave background anisotropies, large-scale structure and primordial nucleosynthesis abundances provide stringent constraints on early-universe physics, allowing us to infer conditions right after the Big Bang. This inferential framework parallels evolutionary biology, where Darwin’s theory of natural selection enables reconstruction of the tree of life from morphological and genetic data. Both cosmology and evolutionary theory transform present-day observations into historically coherent models, demonstrating how systems governed by contingent, path-dependent processes can still be understood through scientific analysis grounded in universal principles. Perhaps it should not be surprising to read from Susskind, a well-known cosmologist, that ‘modern cosmology really began with Darwin and Wallace’ [75]. Similarly, the powerful views of cosmology have inspired others working in biology to build a Big Bang model for life origins and evolution [76].

One source of inspiration with high explanatory power is connected with the physics of phase transitions. This theory, a fundamental pillar of physics, explains how systems undergo abrupt, universal changes driven by small parameter shifts. Its core concepts, symmetry breaking and criticality, apply across scales, from materials to cosmology, revealing deep connections between seemingly unrelated phenomena [77–79]. One of the most striking aspects of the theory of phase transitions is that it derives powerful, often exact results from remarkably simple models, such as the Ising model or percolation lattices [72]. Despite their minimal assumptions, these models capture the essential features of critical behaviour, revealing that the macroscopic properties near phase transitions depend not on microscopic details but on broad features like dimensionality and symmetries. This phenomenon, known as universality, explains why diverse systems, from magnets to fluids to the early universe, exhibit identical critical exponents and scaling laws, highlighting the deep, emergent order underlying complex phenomena.

As discussed in [72], the transition from non-living matter to living matter involves qualitative shifts in system organization, such as symmetry breaking, percolation and bifurcation phenomena, that mirror phase transitions in physical systems. These transitions help explain how relatively simple molecular mixtures, under the right environmental and energetic conditions, could give rise to complex, self-sustaining and evolvable chemical networks. Key processes in early evolution, including the origin of molecular chirality, the appearance of replicators and the onset of cooperation, are framed as critical transitions driven by underlying symmetry changes or threshold effects [80]. This perspective complements traditional chemical and biochemical approaches by offering a theoretical framework based on physics that can describe the nonlinear nature of life’s emergence and the role of collective phenomena in enabling major transitions.

Another approach is to turn to physics itself, since life and the scaling laws it often presents can be rigorously derived as evolution under fundamental physical constraints [81]. For example, classic work on OOL made strong connections with thermodynamics [82],81 ]. Thermodynamics is fundamental to understanding biological complexity because living systems are open, far-from-equilibrium structures that maintain order by dissipating energy. The second law sets constraints on what biological systems can do, while non-equilibrium thermodynamics explains how they sustain gradients, perform work and self-organize. From metabolism to molecular machines, thermodynamic principles reveal how life harnesses energy flows to construct and sustain complex adaptive structures. Within the context of OOL, prebiotic environments provided nonequilibrium conditions, such as thermal gradients, redox potentials or chemical fluxes, that enabled the formation of self-organizing structures [83–86].

Within biology, and particularly with respect to molecular systems, the thermodynamic picture must be consistent with the presence of thresholds [80]. This connection is made in [87], where the author presents an elegant approach to Darwinian dynamics of molecular replicators using a thermodynamic perspective. He shows that modern biomolecules, such as RNA, conform to fundamental bounds that connect fitness and thermodynamics. In particular, it generalizes the classic results by Eigen *et al*. on the error threshold [88], using the thermodynamic formalism. The bounds presented in this article are the type of constraint that should be upheld by life everywhere in the universe.

There is another way to search for potential universal principles by considering purely informational constraints. Information theory plays a central role in theoretical efforts to explain the origins of life, as it provides a formal framework for understanding how order, structure and function can emerge and persist in physical systems far from equilibrium. At its core, life involves the storage, transmission and transformation of information, whether in the replication of nucleic acids, the translation of genetic codes or the regulation of metabolic networks. Shannon’s theory [89] enables researchers to quantify the information content of molecular sequences and to distinguish between randomness and functional complexity [90–92]. Moreover, concepts such as mutual information, entropy and channel capacity have been used to study how early protocells might have maintained and transmitted adaptive information under noisy prebiotic conditions [93].

A remarkable feature of life as we know it is the universal nature of the information material. Every single living cell on our planet contains DNA as the substrate of heritability. Why? Why not a biosphere with multiple forms of molecular information? Perhaps one of the most celebrated universals in biology is the *central dogma* of molecular biology. First articulated by Francis Crick in 1958 [94,95], the central dogma was defined as a principle about the allowed flow of information in biological systems: once information has passed into a protein, it cannot flow back to nucleic acids. This formulation was not merely a description of the transcription–translation pathway but a broader statement about the asymmetry of information transfer in living systems. Within this framework, DNA serves as a stable repository of genetic instructions, which are transcribed into RNA and subsequently translated into proteins, thus linking genotype to phenotype. Later discoveries, including reverse transcription, RNA editing and regulatory roles of noncoding RNAs, have refined, but not overturned, Crick’s central insight [96,97]. This raises deeper questions: Is the structure of information flows described by the central dogma a historical contingency of terrestrial biology, or does it reflect a universal constraint on how any living system must be organized? Theoretical work suggests that the latter may indeed be the case.

In a very elegant theoretical study, Takeuchi & Kaneko demonstrated how the fundamental asymmetries of the central dogma, namely, information flowing from genomes to enzymes and catalysis being performed only by enzymes, could have emerged spontaneously from evolutionary dynamics [98] (see also [99]). Using a mathematical model of protocells containing replicating molecules, the study demonstrates that a trade-off between acting as a catalyst and as a template results in a conflict between molecular and cellular selection. This conflict drives a symmetry breaking, causing molecules to differentiate into genomes (information carriers) and enzymes (catalysts). The process is reinforced by a feedback loop that involves reproductive value and selection strength across different levels. The findings suggest that the central dogma arises naturally from multilevel selection, especially when the molecular-level variation is high compared with the cellular-level variation. In a new paper in this issue [100], the authors propose a generalization of the central dogma using the separation of information from function, which they connect to the broader idea of division of labour. They show that this separation has occurred multiple times throughout the history of life beyond the central dogma, such as with the specialization of germline and somatic cells (a crucial step towards complex multicelularity).

The previous examples involved two important components of life: metabolism and information. A third ingredient in the definition of (cellular) life is the presence of compartments. Compartments have been shown to enhance life in a variety of ways, including speeding up chemical reactions through concentration and increasing evolutionary selection through increased individual fidelity and protection against destabilizing or cheating dynamics in complex reaction networks [6,101–107]. Physical space in modern cells has become a central question in understanding the physiology and biophysics of cells from the constraints of reaction rates, diffusion, packing limits and macromolecular composition [108–111].For example, the smallest cells are nearly completely packed with biomolecules [110]. What consequences do these constraints have for the origin of cellular life? In [107], the authors consider a generalized interplay between function and information, in the context of encapsulation constraints. They show that the complexity of the biochemical cycle and the chemical and biophysical properties of molecules set limits on the possibility of encapsulation and connect this to known constraints on cells owing to scaling relationships for energetic and molecular crowding [110–113].

## 4. Pathways towards life

On a large scale, scientists have established the necessary physical constraints regarding the required planetary conditions for life [26], allowing us to estimate the likelihood of the existence of other Earth-like worlds [114–116] and to establish a catalogue of exoplanets within the habitable zone [117], i.e. where water can exist in a liquid state on the planet’s surface. Assuming that the right physical conditions are met, most of the origin-of-life hypotheses converge on a minimal set of physicochemical requirements [60]. Once again, a universal requirement is the presence of liquid water [118,119], which facilitates molecular motion, reaction kinetics and stable hydrogen bonding. A suitable temperature range, typically between 0°C and 150°C, is also essential to preserve molecular structures [119] (see also [120]). In addition, life requires a diverse chemical inventory, particularly the availability of key biogenic elements, namely carbon, hydrogen, nitrogen, oxygen, phosphorus and sulphur (CHNOPS) [121,122]. These are required to form the basis for essential biomolecules such as nucleic acids, proteins and lipids [60].

Our understanding of the chemical and geochemical details associated with the OOL on Earth has a long history, with commitments to and debates about what conditions are essential [1,73,74,102]. Recently, a variety of specific environments have been proposed as candidates for the evolution of specific aspects of life. These include polymerization of functional and informational polymers in wet/dry cycles [123] or atmospheric aerosols [124] and energy-harnessing membranes in hydrothermal vents [103]. In terms of what is possible, there have been many recent attempts to investigate alternative chemical systems on Earth or other planetary bodies [125,126] and to unravel the detailed history of their biochemical evolutionary history [127–131]. However, a persistent challenge remains: the necessary conditions for these processes often appear incompatible and are seldom found together in a single environment. This has limited the plausibility of models that attempt to explain the emergence of living systems from nonliving matter within a singular, unified setting.

To address this, a broader perspective is emerging, one that frames the OOL within the planetary-scale complexity of the Hadean Earth [132,133]. This view suggests that the diverse and dynamic environments of the early Earth collectively contributed to the emergence of life. Chemically rich microenvironments, though potentially uninhabitable for early life forms, may have provided critical building blocks. Minerals are thought to have served as catalytic surfaces and, at the same time, both local mixing and global transport processes, as well as weathering or geological activity, could have connected spatially separated chemical reactions, enabling a distributed network of prebiotic chemistry. Within this geochemical mosaic, molecular diversity, combined with selective processes in specific locales, probably drove the accumulation and refinement of complex organic molecules required for life [132].

How were the essential components for life listed above mobilized and concentrated in prebiotic environments on the early Earth? In [134], the authors identify planetary processes that probably enabled the availability of these elements in forms suitable for supporting key prebiotic reactions. In this context, terrestrial acidic hot springs and deep-sea hydrothermal vents emerge as particularly favourable settings owing to their potential to drive abiotic nitrogen reduction, release reactive phosphorus compounds and leach key transition metals from igneous rocks. These findings also have broader implications, suggesting that similar geochemical conditions on early Mars and other terrestrial planets could have supported independent pathways to life.

Monod said that ‘every living being is […] a fossil’ [58, ch. 9], which means that the evolutionary past leaves a distinguishable trace within living entities. In some cases, the geochemical context is part of this trace. Can the analysis of cellular structures and cellular networks tell us something about early and alternative scenarios for the emergence of cellular life? An example is provided by the phylogenomic analysis of universal inorganic cellular components [135]. This study supports the conjecture that the first cells might have originated in geothermal environments rich in phosphate and potassium. The crucial concept is that, because early protocells lacked ion pumps and impermeable membranes, their internal chemistry reflected the composition of their surroundings. The analysis suggests that life probably originated in anoxic, metal-enriched geothermal ponds, which have left a fossil trace in the internal composition of modern cells.

Similarly, the study of metabolic networks from an evolutionary perspective offers a highly valuable approach to identify origins [131,136–139]. The first cells emerged and evolved within the context of early Earth as described above, giving rise to the Last Universal Common Ancestor (LUCA). Reconstructing this early metabolic network is key to understanding the transition from prebiotic chemistry to fully functional cellular life. By tracing conserved enzymatic cores and ancient metabolic pathways, we can uncover the robust biochemical logic and environmental constraints that guided early evolution [139,140]. This network serves as a molecular fossil, offering a window into how life first captured energy and processed information within a self-sustaining, organized system.

Are the paths towards life describable as linear chains of events? In [141], a novel way to think about how biosynthetic pathways originated and evolved is proposed, while [142] addresses the deep roots of metabolism before the LUCA. Both contributions converge on a critical distinction in OOL research: the difference between the mere prebiotic availability of metabolic intermediates and the emergence of structured biosynthetic pathways. Although several compounds found in extant metabolisms can indeed form abiotically under prebiotic conditions, this chemical convergence does not by itself imply the existence of energetically coupled, directional pathways. In contrast, biosynthetic pathways are defined by stepwise transformations of a single precursor, enabling energy transduction and regulatory control, characteristics that are absent in a purely chemical network lacking genetic and enzymatic constraints. Together, these works challenge the notion that modern metabolism could have arisen simply through the co-occurrence of metabolites, underscoring the need for mechanisms capable of stabilizing and propagating reaction sequences, such as genetic encoding and selective catalysis.

## 5. Beyond terrestrial life: exobiology, xenobiology and virtual life

In addition to studying potential origins of ‘life as we know it’ on Earth, there are strands of research that study ”life as it could be’. This includes alternative terrestrial life forms obtained through tissue-engineering of living matter (through synthetic biology and xenobiology), possible extraterrestrial life forms (exobiology or astrobiology) and life forms that exist in virtual environments (*in silico* life). This can exploit the possibilities of building, simulating or synthesizing living or lifelike systems that are very different from terrestrial life.

Life elsewhere—extraterrestrial life—could be so different from life on Earth that its underlying principles have nothing to do with our understanding of living matter. This is the problem of ‘life as we don’t know it’ [4,143–145]: can we articulate a universal approach that could safely explain the properties of unknown life? These are important questions, particularly within the growing field of astrobiology [3,34,146–149]. The potential diversity of life forms that could have emerged and evolved elsewhere raises two relevant questions. The first is how we can study potential scenarios for the origins of life using experiments. The second question is: What kinds of scales, case studies, model systems and theoretical approaches can capture the universal properties of the problem?

Returning to Earth, synthetic biology is an engineering discipline that constructs living systems based on modified underlying principles of terrestrial life. However, designs sometimes depart from their technological and biological counterparts [150]. Xenobiology alters the genetic code, expanding the amino acid palette used to construct proteins and introducing novel nucleotides beyond the standard ACGT sequence in DNA, thereby increasing the possibilities for space exploration. In [151], a perspective review of one approach to achieving these xenobiological ends is presented. In an *in vitro* approach, cell-free expression systems (CFS) allow the creation and investigation of biological reactions outside living cells, synthetic pathways, alternative metabolisms, different information replication processes and compartmentalization processes [152]. These ‘roads not taken’ can help provide clues about the deterministic (necessity) versus random (chance), elements of the OOL, and origins of their natural counterparts.

Systems biology techniques can provide valuable insights into the origins of life by enabling researchers to construct simplified, controllable models of early living systems. By reconstructing minimal cells, or protocells, from the bottom up, scientists can test hypotheses about how life-like behaviour could emerge from nonliving components. These experimental platforms help to identify which molecular characteristics and organizational principles are essential for life, explore plausible prebiotic pathways and reveal the conditions under which key transitions, such as replication, metabolism and compartmentalization [153,154], can occur. In essence, synthetic biology turns the question of the OOL into an experimentally tractable problem and allows the incorporation of different scales, from cells to ecosystems [155,156].

Demonstrating what a rich field systems biology in general, and protocells in particular, provide for investigating mechanisms relevant to OOL questions, this special issue includes three articles devoted to different aspects of protocellular research. Das & Rajamani [157] investigate the dynamics of an experimental system that incorporates ecological interactions among protocell populations. They use heterogeneous populations of protocells that have the simpler membranes expected during the OOL. Such membranes can spontaneously self-assemble, and each can have a different chemical makeup. In a population of protocells with different membrane compositions, cells can interact differentially, both synergistically and antagonistically, leading to complex population dynamics. The authors show that heterogeneous systems exhibit emergent growth properties compared with homogeneous systems, suggesting ways in which prebiotic and early biotic diversity could be beneficial. On the theoretical side, these diverse populations and their importance in the OOL are studied in [158] by using a simulation platform as a way to investigate how metabolic regulation can be used to enable simple adaptive or learning behaviours, without the need for genetics. By extending consumer–resource models to include stochastic evolution, individual-level novelty and short-term memory, the study shows that simple, pre-genetic forms of adaptation and learning can significantly affect protocell survival during their lifetimes. The findings highlight the importance of metabolic regulation and agent-like behaviour in shaping the evolutionary dynamics of early life.

Thomsen & Rasmussen [159] take another simulation-based protocell approach to study the molecular mechanisms directly related to metabolism, with results compared with values of wetlab-derived parameters. The simulation enables a detailed examination of the combined fitness of different processes that have opposing requirements. The use of simulation in OOL research has its advantages: experiments can typically be run more readily and the experimenter has ready access to all of the state parameter values at each time step, allowing in-depth analyses. It also has disadvantages, particularly the need to implement adequate underlying physical and chemical mechanisms so that a simulated evolutionary process has sufficiently diverse and powerful underlying properties to exploit, without hard-coding in desired or expected outcomes. Wet-lab and simulation experiments can provide valuable complementary approaches for investigating complex behaviours and emergent properties.

The field of Artificial Life goes beyond synthetic biology’s use of standard or modified biological components to study alternative biochemical systems, and of inorganic systems to investigate various fundamental processes of life. Such studies are relevant both for exobiology (life on other planets that exploits alternative chemical pathways) and to prebiotic processes relevant to origins of life (e.g. crystals and clays [46,49]).

A step further away from terrestrial-type life is to move from the physical (material) to the computational (abstract) domain, into the realm of virtual life. Whether virtual life (in contrast to a mere simulation of material life) is possible depends at least in part on the chosen definition of life. In this regard, however, it does not differ from the possibility of any forms of life that deviate sufficiently from the common instantiation—for example, the possibility of inorganic material life. In [160], Stepney provides a requirement-based definition of life that does not exclude the possibility of virtual life and discusses how a virtual system might fulfil these requirements. Since these requirements are necessarily abstracted away from the specifics of terrestrial life, they provide a different lens on possible origins and on the possibility of partial life that fulfils some but not all of the requirements (see also [161]). For example, artificial life need not originate through an evolutionary process (unless one includes the evolution of the artificer).

## 6. Discussion

Discovering life elsewhere in the universe or creating it in the laboratory would constitute a profound scientific breakthrough, reshaping our understanding of biology, evolution and the uniqueness of life on Earth. In the search for extraterrestrial life, two promising avenues are actively being pursued. The first involves exploring our solar system, particularly environments such as Mars, Europa and Enceladus, where liquid water and geochemical activity can support microbial ecosystems. The second focuses on detecting biosignatures from exoplanets, using next-generation telescopes to identify atmospheric gases or surface features that may indicate biological activity.

By examining the early environments of Earth, the planetary processes and the biochemical systems, and comparing them with what we know about other planets in our solar system and beyond, we can begin to understand the prerequisites for life. Recent discoveries of exoplanets, some within habitable zones with intriguing chemistry [162–164], further challenge us to consider whether life is an inevitable outcome of planetary evolution or an astonishing cosmic fluke. It also pushes us to have better generative theories and experiments to rule in or out particular measurements, to guide and propose new measurements, and to serve as a test bed for exploring ideas.

On the experimental side, efforts to create life in the lab are converging on the challenge of constructing a minimal, self-sustaining and self-replicating protocellular system. This endeavour is inherently multidisciplinary, involving prebiotic chemistry, synthetic biology, systems chemistry and biophysics, as well as philosophy [165]. Multiple potential pathways are under active investigation, each offering complementary insights into how nonliving matter might transition into life [35,166–168]. As discussed in previous sections, these approaches are informed by diverse sources of evidence—geological, biochemical and theoretical—and their integration may ultimately illuminate viable routes to the emergence of life.

**Figure 2.**
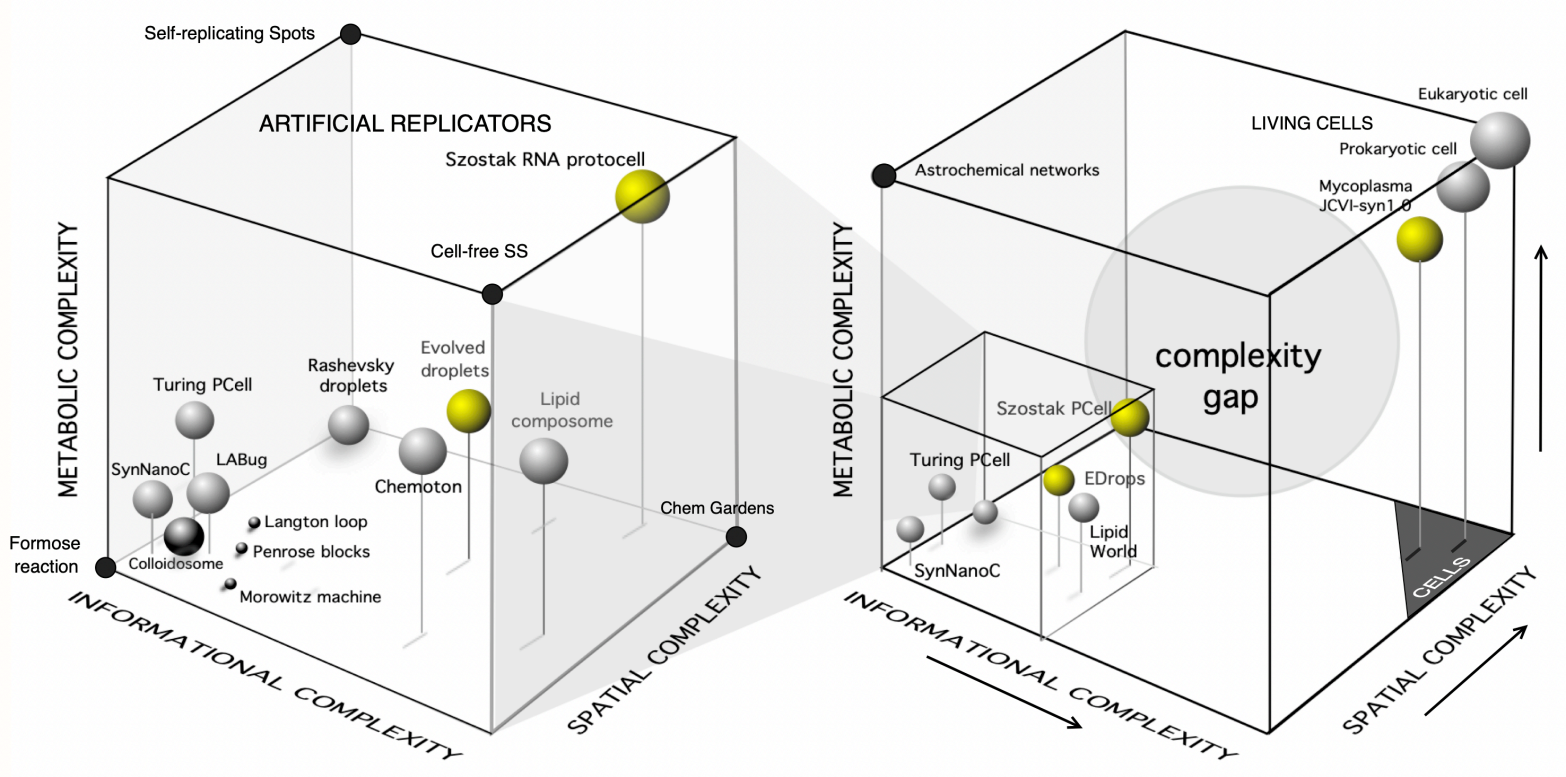
Life as a space of the possible. This figure presents a qualitative morphospace in which three distinct aspects of complexity—related to extant, synthetic and theoretical self-replicating systems—are used to position a range of case studies relative to one another. Included are modern cells, synthetic protocells, minimal-genome cells, theoretical models of self-replicating entities, spatially distributed cellular automata and thermodynamic machines. The right panel shows the entire morphospace, highlighting a large void that may represent either unexplored or fundamentally inaccessible regions. The left panel provides a close-up view of a region populated by synthetic droplets, protocells and various theoretical constructs. Some non-cellular systems are also indicated using black circles as a reference. Within the synthetic cube (left), we indicate: (1) self-replicating spots [171], cell-free systems (SS) [172], lipid composome [173], evolved droplets (EDrops) [35], the chemoton [174], Rashevsky droplets [175], Turing protocells (Turing PCell) [176], synthetic nanocells (SynNanoC) 177], replicating inorganic colloidosomes (black-shaded sphere) [178] or the Los Alamos Bug (LABug) [179]. We also indicate chemical gardens (Chem Gardens) as another corner of spatially extended self-organizing chemical systems [180,181]. Additionally, three classic examples of abstract models of self-reproducing machines or cells are also indicated, namely: (i) Langton's loop [182] based on a cellular automaton running on a two-dimensional lattice, (ii) Penrose blocks [183] based on physical blocks that interact and self-assemble into larger structures and can display ‘reproduction’ and (iii) Morowitz electromechanical machine [184].

What is life then? This question illustrates how hard the theoretical side of the problem is, and typically raises multiple answers [55,169,170]. One way to gain traction on this problem is to define the entire space of possibilities within a category: the space of possible living systems. We have attempted to do this by considering cellular replicators as a subset of an important class of objects that have some degree of embodiment (usually a closed membrane), can store and/or manipulate information, and have a metabolism. In figure 2, we present our morphospace of replicators, defined qualitatively. The location of each case study along the three axes (spatial, informational and metabolic complexity) is relative to other systems. The distance between each example does not define a metric space. On the right, the whole space is depicted, spanning many orders of magnitude from simple autocatalytic reactions (such as the formose reaction [185], lower left corner) to modern cells in the upper right corner. The smaller cube indicates the subset of systems that involve ‘synthetic’ designs, and the details of this space are shown in the cube on the left.

The space of possible replicators serves to define what a replicator is. Any possible embodied system that can exhibit autonomous replication of some kind has a place in the space. There is no single answer, and different kinds of complex replicators coexist within the boundaries of this space. This reflects intrinsic differences in how replication occurs, ranging from the genetically controlled mechanisms present in modern cells to synthetic systems that rely on the coupling between metabolism and membrane deformation.

As our understanding of potential scenarios for the emergence of life advances, we progressively illuminate different regions of the space of possibilities. Current bottom-up approaches to the origins-of-life (OOL) problem have yielded partial, yet highly encouraging, insights. In particular, our knowledge of how diverse interactions between soft matter, protometabolic networks and information-carrying molecules operate is improving rapidly. It is also increasingly clear that explaining the rise of the first cellular replicators will require integrating both evolutionary dynamics and self-organization into theoretical and engineering frameworks [186–190]. In this context, we anticipate that major advances in protocell research may depend on combining evolutionary dynamics with Darwinian selection, an idea strongly supported by the successful evolution of lipid droplets in chemorobotic platforms [35]. The use of morphospaces has also proved valuable since their inception for exploring the limits of the possible. Observed systems tend to cluster along an imaginary axis connecting two opposed vertices, from minimal to maximal cellular complexity. This pattern may suggest that, despite their different origins, the three key components evolve in a synergistic manner. In contrast, empty regions of the morphospace, such as the one shown in figure 2, point to domains of possibilities not occupied by any known system. Why is this so? In some cases, physical or chemical constraints may forbid these regions from being realized. In others, evolutionary processes may have never reached them, perhaps owing to historical contingencies or environmental limitations.

We close with more questions than answers, but that is the nature of science. Whatever the answers are, they will likely emerge at the crossroads of multiple disciplines and may bring unexpected surprises.

## Data Availability

This article has no additional data.

## Footnotes

^1^
Some of the general implications of Assembly Theory are nowadays under discussion, see [38–41].

## References

[1] Benner SA. 2010 Defining life. Astrobiology **10**, 1021–1030. (10.1089/ast.2010.0524) 21162682 PMC3005285

[2] Woese CR. 2004 A new biology for a new century. Microbiol. Mol. Biol. Rev. **68**, 173–186. (10.1128/MMBR.68.2.173-186.2004) 15187180 PMC419918

[3] Kempes CP, Krakauer DC. 2021 The multiple paths to multiple life. J. Mol. Evol. **89**, 415–426. (10.1007/s00239-021-10016-2) 34254169 PMC8318961

[4] Grefenstette N *et al*. 2024 Life as we don’t know it. Astrobiology **24**, 186. (10.1089/ast.2021.0103) 38498819

[5] Walker SI, Davies PCW. 2013 The algorithmic origins of life. J. R. Soc. Interface **10**, 20120869. (10.1098/rsif.2012.0869) 23235265 PMC3565706

[6] Solé R *et al*. 2024 Fundamental constraints to the logic of living systems. Interface Focus **14**, 20240010. (10.1098/rsfs.2024.0010) 39464646 PMC11503024

[7] Langton CG. 1997 Artificial life: an overview. Cambridge, MA: MIT Press. (10.7551/mitpress/1427.001.0001)

[8] Szostak JW. 2012 Attempts to define life do not help to understand the origin of life. J. Biomol. Struct. Dyn. **29**, 599–600. (10.1080/073911012010524998) 22208251 PMC4208307

[9] Oparin A. 1924 Proiskhozhdenie zhizni. Moscow, Russia: Moskovskii Rabochii.

[10] Oparin A. 1938 The origin of life. New York, NY: Macmillan.

[11] Miller SL. 1953 A production of amino acids under possible primitive Earth conditions. Science **117**, 528–529. (10.1126/science.117.3046.528) 13056598

[12] Bada JL, Lazcano A. 2003 Perceptions of science. Prebiotic soup--revisiting the Miller experiment. Science **300**, 745–746. (10.1126/science.1085145) 12730584

[13] Oró J. 1960 Synthesis of adenine from ammonium cyanide. Biochem. Biophys. Res. Commun. **2**, 407–412. (10.1016/0006-291X(60)90138-8)

[14] Oro J. 1961 Mechanism of synthesis of adenine from hydrogen cyanide under possible primitive Earth conditions. Nature **191**, 1193–1194. (10.1038/1911193a0) 13731264

[15] Schopf JW. 2024 Pioneers of origin of life studies—Darwin, Oparin, Haldane, Miller, Oró—and the oldest known records of life. Life **14**, 1345. (10.3390/life14101345) 39459645 PMC11509469

[16] Oró J. 1961 Comets and the formation of biochemical compounds on the primitive Earth. Nature **190**, 389–390. (10.1038/190389a0) 11537542

[17] Oró J, Mills T, Lazcano A. 1991 Comets and the formation of biochemical compounds on the primitive Earth – a review. Orig. Life Evol. Biosph. **21**, 267–277. (10.1007/BF01808302) 11537542

[18] Sagan C. 1994 The search for extraterrestrial life. Sci. Am. **271**, 92–99. (10.1038/scientificamerican1094-92) 11536646

[19] Chyba CF, Thomas PJ, Brookshaw L, Sagan C. 1990 Cometary delivery of organic molecules to the early Earth. Science **249**, 366–373. (10.1126/science.11538074) 11538074

[20] Walker SI. 2017 Origins of life: a problem for physics, a key issues review. Rep. Prog. Phys. **80**, 092601. (10.1088/1361-6633/aa7804) 28593934

[21] Goldenfeld N, Woese C. 2011 Life is physics: evolution as a collective phenomenon far from equilibrium. Annu. Rev. Condens. Matter Phys. **2**, 375–399. (10.1146/annurev-conmatphys-062910-140509)

[22] Chan BWC, Hong Kong. 2019 Lenia: Biology of Artificial Life. ComplexSystems **28**, 251–286. (10.25088/ComplexSystems.28.3.251)

[23] Plantec E, Hamon G, Etcheverry M, Chan BWC, Oudeyer PY, Moulin-Frier C. 2025 Flow-Lenia: Emergent Evolutionary Dynamics in Mass Conservative Continuous Cellular Automata. Artif. Life **31**, 228–248. (10.1162/artl_a_00471) 40298480

[24] Seager S. 2013 Exoplanet habitability. Science **340**, 577–581. (10.1126/science.1232226) 23641111

[25] Takeuchi N, Hogeweg P, Kaneko K. 2017 Conceptualizing the origin of life in terms of evolution. Phil. Trans. R. Soc. A. **375**, 20160346. (10.1098/rsta.2016.0346) 29133445 PMC5686403

[26] Lingam M, Loeb A. 2018 Physical constraints on the likelihood of life on exoplanets. Int. J. Astrobiol. **17**, 116–126. (10.1017/s1473550417000179)

[27] Schwieterman EW *et al*. 2018 Exoplanet biosignatures: a review of remotely detectable signs of life. Astrobiology **18**, 663–708. (10.1089/ast.2017.1729) 29727196 PMC6016574

[28] Walker SI *et al*. 2018 Exoplanet biosignatures: future directions. Astrobiology **18**, 779–824. (10.1089/ast.2017.1738) 29938538 PMC6016573

[29] Solé RV, Munteanu A. 2004 The large-scale organization of chemical reaction networks in astrophysics. Europhys. Lett. **68**, 170–176. (10.1209/epl/i2004-10241-3)

[30] García-Sánchez M, Jiménez-Serra I, Puente-Sánchez F, Aguirre J. 2022 The emergence of interstellar molecular complexity explained by interacting networks. Proc. Natl Acad. Sci. USA **119**, e2119734119. (10.1073/pnas.2119734119) 35867830 PMC9335321

[31] Fernández-Ruz M, Jiménez-Serra I, Aguirre J. 2023 A theoretical approach to the complex chemical evolution of phosphorus in the interstellar medium. Astrophys. J. **956**, 47. (10.3847/1538-4357/acf290)

[32] Fisher T, Janin E, Walker SI. 2025 A complex systems approach to exoplanet atmospheric chemistry: new prospects for ruling out the possibility of alien life as we know it. Planet. Sci. J. **6**, 116. (10.3847/PSJ/adcc27)

[33] Smith TF, Morowitz HJ. 1982 Between history and physics. J. Mol. Evol. **18**, 265–282. (10.1007/BF01734104) 6178836

[34] Chyba CF, Hand KP. 2005 Astrobiology: the study of the living universe. Annu. Rev. Astron. Astrophys. **43**, 31–74. (10.1146/annurev.astro.43.051804.102202)

[35] Gutierrez JMP, Hinkley T, Taylor JW, Yanev K, Cronin L. 2014 Evolution of oil droplets in a chemorobotic platform. Nat. Commun. **5**, 5571. (10.1038/ncomms6571) 25482304 PMC4268700

[36] Points LJ, Taylor JW, Grizou J, Donkers K, Cronin L. 2018 Artificial intelligence exploration of unstable protocells leads to predictable properties and discovery of collective behavior. Proc. Natl Acad. Sci. USA **115**, 885–890. (10.1073/pnas.1711089115) 29339510 PMC5798325

[37] Banzhaf W, Yamamoto L. 2015 Artificial chemistries. Cambridge, MA: MIT Press. (10.7551/mitpress/9780262029438.001.0001)

[38] Jaeger J. 2024 Assembly theory: what it does and what it does not do. J. Mol. Evol. **92**, 87–92. (10.1007/s00239-024-10163-2) 38453740 PMC10978598

[39] Abrahão FS, Hernández-Orozco S, Kiani NA, Tegnér J, Zenil H. 2024 Assembly theory is an approximation to algorithmic complexity based on LZ compression that does not explain selection or evolution. PLoS Complex Syst **1**, e0000014. (10.1371/journal.pcsy.0000014)

[40] Kempes CP, Lachmann M, Iannaccone A, Fricke GM, Chowdhury MR, Walker SI, Cronin L. 2024 Assembly theory and its relationship with computational complexity. arXiv 2406.12176. (10.48550/arXiv.2406.12176) PMC12408342 40918420

[41] Lynch M. 2025 Complexity myths and the misappropriation of evolutionary theory. Proc. Natl Acad. Sci. USA **122**, e2425772122. (10.1073/pnas.2425772122) 40408411 PMC12167962

[42] Marshall SM *et al*. 2021 Identifying molecules as biosignatures with assembly theory and mass spectrometry. Nat. Commun. **12**, 3033. (10.1038/s41467-021-23258-x) 34031398 PMC8144626

[43] Sharma A, Czégel D, Lachmann M, Kempes CP, Walker SI, Cronin L. 2023 Assembly theory explains and quantifies selection and evolution. Nature **622**, 321–328. (10.1038/s41586-023-06600-9) 37794189 PMC10567559

[44] Hazen RM, Burns PC, Cleaves HJ, Downs RT, Krivovichev SV, Wong ML. 2024 Molecular assembly indices of mineral heteropolyanions: some abiotic molecules are as complex as large biomolecules. J. R. Soc. Interface **21**, 20230632. (10.1098/rsif.2023.0632) 38378136 PMC10878807

[45] Walker SI, Mathis C, Marshall S, Cronin L. 2024 Experimentally measured assembly indices are required to determine the threshold for life. J. R. Soc. Interface **21**, 20240367. (10.1098/rsif.2024.0367) 39563496 PMC11576842

[46] Cairns-Smith AG. 1982 Genetic takeover and the mineral origins of life. Cambrdige, UK: Cambridge University Press. (10.1180/claymin.1984.019.1.15)

[47] Cleland CE. 2007 Epistemological issues in the study of microbial life: alternative terran biospheres? Stud. Hist. Philos. Sci. C **38**, 847–861. (10.1016/j.shpsc.2007.09.007) 18053938

[48] Schuster P. 2011 The mathematics of Darwin’s theory of evolution: 1859 and 150 years later. In The mathematics of Darwin’s legacy (eds FC Chalub, JF Rodrigues), pp. 27–66. Basel, Switzerland: Springer. (10.1007/978-3-0348-0122-5_3)

[49] Cairns-Smith AG. 1966 The origin of life and the nature of the primitive gene. J. Theor. Biol. **10**, 53–88. (10.1016/0022-5193(66)90178-0) 5964688

[50] Pross A. 2011 Toward a general theory of evolution: extending Darwinian theory to inanimate matter. J. Syst. Chem. **2**, 1. (10.1186/1759-2208-2-1)

[51] Byers S. 2006 Life as ‘self-motion’: Descartes and ‘the Aristotelians’ on the soul as the life of the body. Rev. Metaphys **59**, 723–755. https://www.jstor.org/stable/20130699

[52] Cannon WB. 1929 Organization for physiological homeostasis. Physiol. Rev. **9**, 399–431. (10.1152/physrev.1929.9.3.399)

[53] Maturana HR, Varela FJ. 1980 Autopoeisis and cognition: the realization of the living. Dordretch, Holland: D. Reidel. (10.1186/1759-2208-2-1)

[54] Lachmann M, Walker S. 2019 Life!=alive. https://aeon.co/essays/what-can-schrodingers-cat-say-about-3d-printers-on-mars.

[55] Bender R, Kofman K, Arcas BA, Levin M. 2025 What lives? A meta-analysis of diverse opinions on the definition of life. arXiv 250515849. (10.48550/arXiv.2505.15849)

[56] Smith E, Kubica A. 2025 Science of the gaps. Phil. Trans. R. Soc. B **380**, 20240282. (10.1098/rstb.2024.0282) 41035331 PMC12489511

[57] de Duve C. 2011 Life as a cosmic imperative? Phil. Trans. R. Soc. A **369**, 620–623. (10.1098/rsta.2010.0312) 21220285

[58] Monod J. 1971 Chance and necessity. New York, NY: Collins.

[59] Hazen RM. 2017 Chance, necessity and the origins of life: a physical sciences perspective. Phil. Trans. R. Soc. A **375**, 20160353. (10.1098/rsta.2016.0353) 29133451 PMC5686409

[60] Deamer D. 2011 First life: discovering the connections between stars, cells, and how life began. Berkeley, CA: University of California Press. (10.1525/9780520948952)

[61] Johansen A, Sornette D. 2001 Finite-time singularity in the dynamics of the world population, economic and financial indices. Phys. A **294**, 465–502. (10.1016/S0378-4371(01)00105-4)

[62] Hanel R, Kauffman SA, Thurner S. 2005 Phase transitions in random catalytic networks. Phys. Rev. E **72**, 036117. (10.1103/PhysRevE.72.036117) 16241525

[63] Youn H, Strumsky D, Bettencourt LMA, Lobo J. 2015 Invention as a combinatorial process: evidence from US patents. J. R. Soc. Interface **12**, 20150272. (10.1098/rsif.2015.0272) 25904530 PMC4424706

[64] Solé R, Amor DR, Valverde S. 2016 On singularities and black holes in combination-driven models of technological innovation networks. PLoS One **11**, e0146180. (10.1371/journal.pone.0146180) 26821277 PMC4731471

[65] Kauffman SA. 2000 Investigations. New York, NY: Oxford University Press. (10.1093/oso/9780195121049.001.0001)

[66] Wolfram S. 1984 Cellular automata as models of complexity. Nature New Biol. **311**, 419–424. (10.1038/311419a0)

[67] Wolfram S. 1985 Undecidability and intractability in theoretical physics. Phys. Rev. Lett **54**, 735–738. (10.1103/PhysRevLett.54.735) 10031602

[68] Wolfram S. 2002 A new kind of science. Champaign, IL: Wolfram Media.

[69] Ilachinski A. 2001 Cellular automata: a discrete universe. Singapore: World Scientific. (10.1142/4702)

[70] Israeli N, Goldenfeld N. 2004 Computational irreducibility and the predictability of complex physical systems. Phys. Rev. Lett. **92**, 074105. (10.1103/PhysRevLett.92.074105) 14995857

[71] Kauffman SA, Roli A. 2025 Is the emergence of life and of agency expected? Phil. Trans. R. Soc. B **380**, 20240283. (10.1098/rstb.2024.0283) 41035328 PMC12489499

[72] Solé R, de Domenico M. 2025 Bifurcations and phase transitions in the origins of life. Phil. Trans. R. Soc. B **380**, 20240295. (10.1098/rstb.2024.0295) 41035327 PMC12489512

[73] Pace NR. 2001 The universal nature of biochemistry. Proc. Natl Acad. Sci. USA **98**, 805–808. (10.1073/pnas.98.3.805) 11158550 PMC33372

[74] Bains W. 2004 Many chemistries could be used to build living systems. Astrobiology **4**, 137–167. (10.1089/153110704323175124) 15253836

[75] Susskind L. 2008 The cosmic landscape: string theory and the illusion of intelligent design. New York, NY: Back Bay Books. (10.1063/1.2218558)

[76] Koonin EV. 2007 The biological big bang model for the major transitions in evolution. Biol. Direct **2**, 21. (10.1186/1745-6150-2-21) 17708768 PMC1973067

[77] Stanley HE. 1971 Introduction to phase transitions and critical phenomena. New York, NY: Oxford University Press.

[78] Goldenfeld N. 1992 Lectures on phase transitions and the renormalization group. Reading, MA: Addison-Wesley.

[79] Solé R. 2011 Phase transitions. Princeton, NJ: Princeton University Press.

[80] Jeancolas C, Malaterre C, Nghe P. 2020 Thresholds in origin of life scenarios. iScience **23**, 101756. (10.1016/j.isci.2020.101756) 33241201 PMC7674518

[81] Kempes CP, Koehl MAR, West GB. 2019 The scales that limit: the physical boundaries of evolution. Front. Ecol. Evol. **7**, 242. (10.3389/fevo.2019.00242)

[82] Schrödinger E. 1944 What is life? the physical aspect of the living cell. Cambridge, UK: Cambridge University Press.

[83] Morowitz HJ. 1968 Energy flow in biology: biological organization as a problem in thermal physics. New York, NY: Academic Press.

[84] Morowitz H, Smith E. 2007 Energy flow and the organization of life. Complexity **13**, 51–59. (10.1002/cplx.20191)

[85] Smith E. 2008 Thermodynamics of natural selection I: Energy flow and the limits on organization. J. Theor. Biol. **252**, 185–197. (10.1016/j.jtbi.2008.02.010) 18367210

[86] Smith E. 2008 Thermodynamics of natural selection II: Chemical Carnot cycles. J. Theor. Biol. **252**, 198–212. (10.1016/j.jtbi.2008.02.008) 18367209

[87] Kolchinsky A. 2025 Thermodynamics of Darwinian selection in molecular replicators. Phil. Trans. R. Soc. B **380**, 20240436. (10.1098/rstb.2024.0436) 41035319 PMC12489508

[88] Eigen M, Gardiner W, Schuster P, Winkler-Oswatitsch R. 1981 The origin of genetic information. Sci. Am. **244**, 88–92. (10.1038/scientificamerican0481-88) 6164094

[89] Ash RB. 1965 Information theory. New York, NY: Dover.

[90] Küppers BO. 1990 Information and the origin of life. Cambridge, MA: MIT Press.

[91] Adami C. 2004 Information theory in molecular biology. Phys. Life Rev. **1**, 3–22. (10.1016/j.plrev.2004.01.002)

[92] Walker SI, Ellis GF. 2017 The informational architecture of the cell: a systems view. Phil. Trans. R. Soc. 20160392.

[93] Farnsworth KD, Jaeger J. 2013 Living through downward causation: from molecules to ecosystems. Interface Focus **2**, 20130062.

[94] Crick FH. 1958 On protein synthesis. Symp. Soc. Exp. Biol. **12**, 138–163. 13580867

[95] Crick F. 1970 Central dogma of molecular biology. Nature **227**, 561–563. (10.1038/227561a0) 4913914

[96] Thieffry D, Sarkar S. 1998 Forty years under the central dogma. Trends Biochem. Sci. **23**, 312–316. (10.1016/s0968-0004(98)01244-4) 9757833

[97] Ille AM, Lamont H, Mathews MB. 2022 The central dogma revisited: Insights from protein synthesis, CRISPR, and beyond. WIREs RNA **13**, e1718. (10.1002/wrna.1718) 35199457

[98] Takeuchi N, Kaneko K. 2019 The origin of the central dogma through conflicting multilevel selection. Proc. R. Soc. B **286**, 20191359. (10.1098/rspb.2019.1359) PMC6790754 31575361

[99] Takeuchi N, Hogeweg P, Kaneko K. 2017 The origin of a primordial genome through spontaneous symmetry breaking. Nat. Commun. **8**, 250. (10.1038/s41467-017-00243-x) 28811464 PMC5557888

[100] Takeuchi N, Kaneko K. 2025 Generalising the central dogma as a cross-hierarchical principle of biology. Phil. Trans. R. Soc. B 20240296. (10.1098/rstb.2024.0296) 41035325 PMC12489501

[101] Plum AM, Kempes CP, Peng Z, Baum DA. 2025 Spatial structure supports diversity in prebiotic autocatalytic chemical ecosystems. Npj Complex. **2**. (10.1038/s44260-025-00045-z) PMC12226336 40621055

[102] Morowitz HJ, Smith E. 2016 The origin and nature of life on Earth: the emergence of the fourth geosphere. New York, NY: Cambridge University Press. (10.1017/CBO9781316348772)

[103] Lane N, Martin WF. 2012 The origin of membrane bioenergetics. Cell **151**, 1406–1416. (10.1016/j.cell.2012.11.050) 23260134

[104] Takeuchi N, Hogeweg P. 2009 Multilevel selection in models of prebiotic evolution II: a direct comparison of compartmentalization and spatial self-organization. PLoS Comput. Biol. **5**, e1000542. (10.1371/journal.pcbi.1000542) 19834556 PMC2757730

[105] Saha R, Pohorille A, Chen IA. 2014 Molecular crowding and early evolution. Orig. Life Evol. Biosph. **44**, 319–324. (10.1007/s11084-014-9392-3) 25585804

[106] Wächtershäuser G. 1988 Before enzymes and templates: theory of surface metabolism. Microbiol. Rev. **52**, 452–484. (10.1128/mr.52.4.452-484.1988) 3070320 PMC373159

[107] Kempes C, Avilla D, Mathis C. 2025 How hard is it to encapsulate life? The general constraints on encapsulation. Phil. Trans. R. Soc. B **380**, 20240297. (10.1098/rstb.2024.0297) 41035324 PMC12489500

[108] Rivas G, Herzfeld J. 2004 Life in a crowded world: workshop on the biological implications of macromolecular crowding. EMBO **5**, 23–27. (10.1038/sj.embor.7400056) PMC1298967 14710181

[109] Zhou HX, Rivas G, Minton AP. 2008 Macromolecular crowding and confinement: biochemical, biophysical, and potential physiological consequences. Annu. Rev. Biophys. **37**, 375–397. (10.1146/annurev.biophys.37.032807.125817) 18573087 PMC2826134

[110] Kempes CP, Wang L, Amend JP, Doyle J, Hoehler T. 2016 Evolutionary tradeoffs in cellular composition across diverse bacteria. ISME J. **10**, 2145–2157. (10.1038/ismej.2016.21) 27046336 PMC4989312

[111] Ritchie ME, Kempes CP. 2023 Metabolic scaling in small life forms. bioRxiv 2023.12.20.572702. (10.1101/2023.12.20.572702)

[112] Kempes CP, Dutkiewicz S, Follows MJ. 2012 Growth, metabolic partitioning, and the size of microorganisms. Proc. Natl Acad. Sci. USA **109**, 495–500. (10.1073/pnas.1115585109) 22203990 PMC3258615

[113] Kempes CP, van Bodegom PM, Wolpert D, Libby E, Amend J, Hoehler T. 2017 Drivers of bacterial maintenance and minimal energy requirements. Front. Microbiol. **8**, 31. (10.3389/fmicb.2017.00031) 28197128 PMC5281582

[114] Anglada-Escudé G *et al*. 2016 A terrestrial planet candidate in a temperate orbit around Proxima Centauri. Nature **536**, 437–440. (10.1038/nature19106) 27558064

[115] Dong C, Jin M, Lingam M, Airapetian VS, Ma Y, van der Holst B. 2018 Atmospheric escape from the TRAPPIST-1 planets and implications for habitability. Proc. Natl Acad. Sci. USA **115**, 260–265. (10.1073/pnas.1708010115) 29284746 PMC5777028

[116] Madhusudhan N. 2019 Exoplanetary atmospheres: key insights, challenges, and prospects. Annu. Rev. Astron. Astrophys. **57**, 617–663. (10.1146/annurev-astro-081817-051846)

[117] Hill ML, Bott K, Dalba PA, Fetherolf T, Kane SR, Kopparapu R, Li Z, Ostberg C. 2023 A catalog of habitable zone exoplanets. Astron. J. **165**, 34. (10.3847/1538-3881/aca1c0)

[118] Ball P. 2001 Life’s matrix: a biography of water. Berkeley, CA: University of California Press.

[119] Westall F, Brack A. 2018 The importance of water for life. Space Sci. Rev. **214**, 50. (10.1007/s11214-018-0476-7)

[120] do Nascimento Vieira A, Kleinermanns K, Martin WF, Preiner M. 2020 The ambivalent role of water at the origins of life. FEBS Lett. **594**, 2717–2733. (10.1002/1873-3468.13815) 32416624

[121] Williams RJP. 1991 The chemical elements of life. J. Chem. Soc. 539–546. (10.1039/dt9910000539)

[122] Silva JF, Williams RJP. 2001 The biological chemistry of the elements: the inorganic chemistry of life. Oxford, UK: Oxford University Press. (10.1093/oso/9780198508472.001.0001)

[123] Damer B, Deamer D. 2020 The hot spring hypothesis for an origin of life. Astrobiology **20**, 429–452. (10.1089/ast.2019.2045) 31841362 PMC7133448

[124] Dobson CM, Ellison GB, Tuck AF, Vaida V. 2000 Atmospheric aerosols as prebiotic chemical reactors. Proc. Natl Acad. Sci. USA **97**, 11864–11868. (10.1073/pnas.200366897) 11035775 PMC17260

[125] Goldford JE, Hartman H, Smith TF, Segrè D. 2017 Remnants of an ancient metabolism without phosphate. Cell **168**, 1126–1134. (10.1016/j.cell.2017.02.001) 28262353

[126] Smith HB, Drew A, Malloy JF, Walker SI. 2021 Seeding biochemistry on other worlds: Enceladus as a case study. Astrobiology **21**, 177–190. (10.1089/ast.2019.2197) 33064954 PMC7876360

[127] Petrov AS *et al*. 2014 Evolution of the ribosome at atomic resolution. Proc. Natl Acad. Sci. USA **111**, 10251–10256. (10.1073/pnas.1407205111) 24982194 PMC4104869

[128] Petrov AS *et al*. 2015 History of the ribosome and the origin of translation. Proc. Natl Acad. Sci. USA **112**, 15396–15401. (10.1073/pnas.1509761112) 26621738 PMC4687566

[129] Goldman AD, Kacar B. 2021 Cofactors are remnants of life’s origin and early evolution. J. Mol. Evol. **89**, 127–133. (10.1007/s00239-020-09988-4) 33547911 PMC7982383

[130] Garcia AK, Kaçar B. 2019 How to resurrect ancestral proteins as proxies for ancient biogeochemistry. Free Radic. Biol. Med. **140**, 260–269. (10.1016/j.freeradbiomed.2019.03.033) 30951835

[131] Kaçar B. 2024 Reconstructing early microbial life. Annu. Rev. Microbiol. **78**, 463–492. (10.1146/annurev-micro-041522-103400) 39163590

[132] Stüeken EE, Anderson RE, Bowman JS, Brazelton WJ, Colangelo-Lillis J, Goldman AD, Som SM, Baross JA. 2013 Did life originate from a global chemical reactor? Geobiology **11**, 101–126. (10.1111/gbi.12025) 23331348

[133] Kitadai N, Maruyama S. 2018 Origins of building blocks of life: a review. Geosci. Front. **9**, 1117–1153. (10.1016/j.gsf.2017.07.007)

[134] Galloway T, Cousins CR, Baidya A, Stüeken EE. 2025 Planetary sources of bio-essential nutrients on a prebiotic world. Phil. Trans. R. Soc. B **380**, 20240288. (10.1098/rstb.2024.0288) 41035321 PMC12489510

[135] Mulkidjanian AY, Bychkov Ay, Dibrova DV, Galperin MY, Koonin EV. 2012 Origin of first cells at terrestrial, anoxic geothermal fields. Proc. Natl Acad. Sci. USA **109**, E821–E830. (10.1073/pnas.1117774109) 22331915 PMC3325685

[136] Preiner M *et al*. 2018 Serpentinization: connecting geochemistry, ancient metabolism and industrial hydrogenation. Life **8**, 41. (10.3390/life8040041) 30249016 PMC6316048

[137] Xavier JC, Kauffman S. 2022 Small-molecule autocatalytic networks are universal metabolic fossils. Phil. Trans. R. Soc. A. **380**, 20210244. (10.1098/rsta.2021.0244) 35599556

[138] Harrison SA, Rammu H, Liu F, Halpern A, Nunes Palmeira R, Lane N. 2023 Life as a Guide to Its Own Origins. Annu. Rev. Ecol. Evol. Syst. **54**, 327–350. (10.1146/annurev-ecolsys-110421-101509)

[139] Mrnjavac N, Schwander L, Brabender M, Martin WF. 2024 Chemical antiquity in metabolism. Acc. Chem. Res. **57**, 2267–2278. (10.1021/acs.accounts.4c00226) 39083571 PMC11339923

[140] Weiss MC, Preiner M, Xavier JC, Zimorski V, Martin WF. 2018 The last universal common ancestor between ancient Earth chemistry and the onset of genetics. PLoS Genet. **14**, e1007518. (10.1371/journal.pgen.1007518) 30114187 PMC6095482

[141] Negrón-Mendoza AHM, Hernández-Morales R, Lazcano A. 2025 Can the origin of biosynthetic routes be explained by a Frankenstein’s monster-like spontaneous assembly of prebiotic reactants? Phil. Trans. R. Soc. B **380**, 20240289. (10.1098/rstb.2024.0289) 41035326 PMC12489505

[142] Carbonell P, Pereto J. 2025 Before LUCA: unearthing the chemical roots of metabolism. Phil. Trans. of Soc. B **380**, 20240292. (10.1098/rstb.2024.0292) PMC12489507 41035332

[143] Wächtershäuser G. 2000 Life as we don’t know it. Science **289**, 1307–1308. (10.1126/science.289.5483.1307) 10979855

[144] Cleland CE. 2019 The quest for a universal theory of life: searching for life as we don’t know it. Cambridge, UK: Cambridge University Press. (10.1017/9781139046893)

[145] Walker SI. 2024 Life as no one knows it: the physics of life’s emergence. New York, NY: Penguin.

[146] Schopf JW. 1999 Cradle of life: the discovery of earth’s earliest fossils. Princetom, NJ: Princeton University Press. (10.1063/1.882868)

[147] Des Marais DJ, Walter MR. 1999 Astrobiology: exploring the origins, evolution, and distribution of life in the Universe. Annu. Rev. Ecol. Syst. **30**, 397–420. (10.1146/annurev.ecolsys.30.1.397) 11543275

[148] Smith HH *et al*. 2021 The grayness of the origin of life. Life **11**, 498. (10.3390/life11060498) 34072344 PMC8226951

[149] Asche S. 2023 What it takes to solve the origin (s) of life: an integrated review of techniques. arXiv 2308.11665. (10.48550/arXiv.2308.11665)

[150] Solé RV, Macia J. 2013 Expanding the landscape of biological computation with synthetic multicellular consortia. Nat. Comput. **12**, 485–497. (10.1007/s11047-013-9380-y)

[151] Hofmann M, Abdo F, Borkowski O, Kushwaha M. 2025 Reconstituting alternative life using the test-bed of cell-free systems. Phil. Trans. R. Soc. B **380**, 20240293. (10.1098/rstb.2024.0293) 41035330 PMC12489509

[152] Noireaux V, Liu AP. 2020 The New Age of Cell-Free Biology. Annu. Rev. Biomed. Eng. **22**, 51–77. (10.1146/annurev-bioeng-092019-111110) 32151150

[153] Gánti T. 2003 Chemoton theory vol 2: theory of living systems. New York, NY: Plenum.

[154] Solé RV, Munteanu A, Rodriguez-Caso C, Macía J. 2007 Synthetic protocell biology: from reproduction to computation. Phil. Trans. R. Soc. B **362**, 1727–1739. (10.1098/rstb.2007.2065) 17472932 PMC2442389

[155] Solé R, Maull V, Amor DR, Mauri JP, Núria CP. 2024 Synthetic ecosystems: from the test tube to the biosphere. ACS Synth. Biol. **13**, 3812–3826. (10.1021/acssynbio.4c00384) 39570594 PMC11669164

[156] Maull V, Pla Mauri J, Conde Pueyo N, Solé R. 2024 A synthetic microbial Daisyworld: planetary regulation in the test tube. J. R. Soc. Interface **21**, 20230585. (10.1098/rsif.2023.0585) 38321922 PMC10847846

[157] Das S, Pal R, Rajamani S. 2025 Dynamical interactions among protocell populations: implications for membrane-mediated chemical evolution. Phil. Trans. R. Soc. B **380**, 20250104. (10.1098/rstb.2025.0104) 41035320 PMC12489506

[158] Shirt-Ediss B, Ferrero-Fernández A, De Martino D, Bich L, Moreno A, Ruiz-Mirazo K. 2025 Modelling the prebiotic origins of regulation and agency in evolving protocell ecologies. Phil. Trans. R. Soc. B **380**, 20240287. (10.1098/rstb.2024.0287) 41035323 PMC12489503

[159] Reinholt Thomsen K, Kolchinsky K, Rasmussen S. 2025 Protocellular energy transduction, information, and fitness. Phil. Trans. R. Soc. B **380**, 20240294. (10.1098/rstb.2024.0294) 41035329 PMC12489498

[160] Stepney S. 2025 Towards origins of virtual artificial life: an overview. Phil. Trans R. Soc. B **380**, 20240298. (10.1098/rstb.2024.0298) 41035322 PMC12489504

[161] Bartlett S, Wong ML. 2020 Defining Lyfe in the universe: from three privileged functions to four pillars. Life **10**, 42. (10.3390/life10040042) 32316364 PMC7235751

[162] Kane SR *et al*. 2016 A catalog of Kepler habitable zone exoplanet candidates. Astrophys. J. **830**, 1. (10.3847/0004-637X/830/1/1)

[163] Bohl A, Lowry G. 2025 Probing the limits of habitability: a catalog of rocky exoplanets in the habitable zone. arXiv arXiv.2501.14054. (10.48550/arXiv.2501.14054)

[164] Madhusudhan N, Constantinou S, Holmberg M, Sarkar S, Piette AAA, Moses JI. 2025 New Constraints on DMS and DMDS in the Atmosphere of K2-18 b from JWST MIRI. Astrophys. J. Lett. **983**, L40. (10.3847/2041-8213/adc1c8)

[165] Bedau MA. 2018 The nature of life. Cambridge University Press.

[166] Henson A, Gutierrez JMP, Hinkley T, Tsuda S, Cronin L. 2015 Towards heterotic computing with droplets in a fully automated droplet-maker platform. Phil. Trans. R. Soc. A **373**, 20140221. (10.1098/rsta.2014.0221) 26078348

[167] Vincent L, Berg M, Krismer M, Saghafi SS, Cosby J, Sankari T, Vetsigian K, Ii HJC, Baum DA. 2019 Chemical ecosystem selection on mineral surfaces reveals long-term dynamics consistent with the spontaneous emergence of mutual catalysis. Life **9**, 80. (10.3390/life9040080) 31652727 PMC6911371

[168] Baum DA, Vetsigian K. 2017 An Experimental framework for generating evolvable chemical systems in the laboratory. Orig. Life Evol. Biospheres **47**, 481–497. (10.1007/s11084-016-9526-x) PMC5705744 27864699

[169] Sagan C, Bedau MA, Cleland CE. 2010 Definitions of life. In The nature of life: classical and contemporary perspectives from philosophy and science (eds MA Bedau, CE Cleland), pp. 303–306. New York, NY: Cambridge University Press. (10.1017/CBO9780511730191.029)

[170] Knuuttila T, Loettgers A. 2017 What are definitions of life good for? Transdisciplinary and other definitions in astrobiology. Biol. Philos. **32**, 1185–1203. (10.1007/s10539-017-9600-4)

[171] Lee KJ, McCormick WD, Pearson JE, Swinney HL. 1994 Experimental observation of self-replicating spots in a reaction–diffusion system. Nature **369**, 215–218. (10.1038/369215a0)

[172] Hodgman CE, Jewett MC. 2012 Cell-free synthetic biology: thinking outside the cell. Metab. Eng. **14**, 261–269. (10.1016/j.ymben.2011.09.002) 21946161 PMC3322310

[173] Lancet D, Zidovetzki R, Markovitch O. 2018 Systems protobiology: origin of life in lipid catalytic networks. J. R. Soc. Interface **15**, 20180159. (10.1098/rsif.2018.0159) 30045888 PMC6073634

[174] Gánti T. 2003 Chemoton theory: theory of living systems. Berlin, Germany: Springer.

[175] Macía J, Solé RV. 2007 Protocell self-reproduction in a spatially extended metabolism-vesicle system. J. Theor. Biol. **245**, 400–410. (10.1016/j.jtbi.2006.10.021) 17184796

[176] Macía J, Solé RV. 2007 Synthetic Turing protocells: vesicle self-reproduction through symmetry-breaking instabilities. Phil. Trans. R. Soc. B **362**, 1821–1829. (10.1098/rstb.2007.2074) 17510018 PMC2442396

[177] Fellermann H, Solé RV. 2007 Minimal model of self-replicating nanocells: a physically embodied information-free scenario. Phil. Trans. R. Soc. B **362**, 1803–1811. (10.1098/rstb.2007.2072) 17510020 PMC2442394

[178] Li M, Huang X, Mann S. 2014 Spontaneous growth and division in self‐reproducing inorganic colloidosomes. Small **10**, 3291–3298. (10.1002/smll.201400639) 24861579

[179] Fellermann H, Rasmussen S, Ziock HJ, Solé RV. 2007 Life cycle of a minimal protocell--a dissipative particle dynamics study. Artif. Life **13**, 319–345. (10.1162/artl.2007.13.4.319) 17716015

[180] Barge LM *et al*. 2015 From chemical gardens to chemobrionics. Chem. Rev. **115**, 8652–8703. (10.1021/acs.chemrev.5b00014) 26176351

[181] Cardoso SSS *et al*. 2020 Chemobrionics: from self-assembled material architectures to the origin of life. Artif. Life **26**, 315–326. (10.1162/artl_a_00323) 32697160

[182] Langton CG. 1986 Studying artificial life with cellular automata. Phys. D **22**, 120–149. (10.1016/0167-2789(86)90237-X)

[183] Penrose LS. 1959 Self-Reproducing Machines. Sci. Am. **200**, 105–114. (10.1038/scientificamerican0659-105)

[184] Morowitz HJ. 1959 A model of reproduction. Am. Sci. **47**, 261–263.

[185] Szathmáry E. 2000 The evolution of replicators. Phil. Trans. R. Soc. B **355**, 1669–1676. (10.1098/rstb.2000.0730) 11127914 PMC1692888

[186] Solé RV. 2009 Evolution and self-assembly of protocells. Int. J. Biochem. Cell Biol. **41**, 274–284. (10.1016/j.biocel.2008.10.004) 18951997

[187] Stano P, Luisi PL. 2010 Achievements and open questions in the self-reproduction of vesicles and synthetic minimal cells. Chem. Commun. **46**, 3639. (10.1039/b913997d) 20442914

[188] Noireaux V, Maeda YT, Libchaber A. 2011 Development of an artificial cell, from self-organization to computation and self-reproduction. Proc. Natl Acad. Sci. USA **108**, 3473–3480. (10.1073/pnas.1017075108) 21317359 PMC3048108

[189] Xu C, Hu S, Chen X. 2016 Artificial cells: from basic science to applications. Mater. Today **19**, 516–532. (10.1016/j.mattod.2016.02.020) PMC5222523 28077925

[190] Hirschi S, Ward TR, Meier WP, Müller DJ, Fotiadis D. 2022 Synthetic biology: bottom-up assembly of molecular systems. Chem. Rev. **122**, 16294–16328. (10.1021/acs.chemrev.2c00339) 36179355

